# Are the Fathers Alright? A Systematic and Critical Review of Studies on Gay and Bisexual Fatherhood

**DOI:** 10.3389/fpsyg.2017.01636

**Published:** 2017-09-21

**Authors:** Francis A. Carneiro, Fiona Tasker, Fernando Salinas-Quiroz, Isabel Leal, Pedro A. Costa

**Affiliations:** ^1^William James Center for Research, Instituto Universitário de Ciências Psicológicas, Sociais e da Vida Lisbon, Portugal; ^2^Department of Psychological Sciences, Birkbeck University of London London, United Kingdom; ^3^National Pedagogic University Mexico Mexico City, Mexico

**Keywords:** gay fathers, bisexual fathers, parenting pathways, degendered parenting, non-traditional families, modern families

## Abstract

The purpose of the present systematic and critical review was to assess the findings and to identify the gaps in the literature concerning gay and bisexual fathers. A comprehensive search of relevant literature using electronic databases and reference lists for articles published until December 2016 was conducted. A total of 63 studies, spanning from 1979 to 2016, were collected. More than half of the studies were published after 2011 and the overwhelming majority were conducted in the United States. Nine themes were identified in the studies reviewed: (1) Pathways to fatherhood; (2) Motivations for fatherhood; (3) Parenting experiences and childrearing; (4) Family life and relationship quality; (5) Gender and father identities and gender-role orientation; (6) Disclosure of sexual identity; (7) Social climate; (8) Father's psychosocial adjustment; and (9) Children's psychosocial adjustment. It was found that research on gay fatherhood appears to be more heterogeneous than on lesbian motherhood, perhaps because of the variety of pathways to parenthood (via co-parenting, adoption, fostering, or surrogacy). Two-father families are becoming more visible in research on sexual minority parenting and gradually transforming the conceptualization of parenting in family research.

## Introduction

Research on families headed by gay/lesbian parents started in the late 1970's, after important milestones in gender equality had been accomplished by the feminist and the gay liberation movements (Patterson, 1992). In the psychological field, a major accomplishment for gay men and lesbians was the removal of homosexuality from the American Psychiatric Association's Diagnostic and Statistical Manual of Mental Disorders in 1973 (DSM; Dresher, 2012). The first empirical articles about gay/lesbian parents were published in scientific journals in the late 1970's, when interest in the outcomes of lesbian and gay parenthood began to increase because of custody disputes regarding children whose mothers (and to a lesser extent, fathers) had come out as gay (Patterson, 1997). After almost 40 years of research, there is still controversy over the effects for children growing up with a gay or a lesbian parent, and several western countries still prohibit same-gender couples' access to alternative routes to parenting based on the argument that these family configurations may hinder normative child development (Fedewa et al., 2015; Takács et al., 2016).

With easier access to donor insemination, many lesbian and bisexual women became mothers either via clinical-based reproductive technologies or self-insemination with donated semen. The exponential growth of lesbian couples and single lesbians becoming mothers in the 1990's was termed the *Lesbian Baby Boom* (Patterson, 1992), and research on lesbian and gay families followed this trend with few studies of gay fatherhood (Biblarz and Savci, 2010).

Gay men have become parents within heterosexual relationships, and through semen donation, surrogacy, step- and co-parenting, adoption, and fostering (Barrett and Tasker, 2001). While there are various routes to parenthood for gay men, most options are largely dependent on State legislation; Same-gender adoption, fostering, or surrogacy are not available to same-gender couples in most European countries (Commissioner for Human Rights, 2011). For example, it is estimated that in Portugal and Italy the overwhelming majority of gay fathers have become parents within heterosexual relationships (Lelleri et al., 2008; Baiocco et al., 2014; Costa and Bidell, 2017). In contrast, the availability of surrogacy and adoption throughout most of North America have opened new fields of scientific enquiry (Gates et al., 2007; Bergman et al., 2010).

Previous reviews on same-gender parenting have only marginally included gay parented families. Among the 23 articles reviewed by Anderssen et al. (2002), only three included gay-father families. Among the 19 studies in Crowl et al. (2008) meta-analysis, no assessment was made of the differences and similarities between gay/lesbian parenting couples, while in Fedewa et al. (2015) meta-analysis, only four studies included gay fathers from a total of 33 reviewed studies. The imbalance in studies on gay and lesbian parented families is important: While there appears to be few overall differences between different-gender and same-gender couples, parental gender or even the interaction between parental gender and sexual orientation may influence parenting practices and family dynamics (Crowl et al., 2008; Bibarz and Stacey, 2010). For example, it has been suggested that gay couples may share household and parenting duties more equally than heterosexual couples, but less equally than lesbian couples. Furthermore, gay couples may bring up less gender traditional children than heterosexual couples, but be more traditional than lesbian couples in their gender socialization of their children (Bibarz and Stacey, 2010).

In addition, there are gender of target effects on the social perception of same-gender couples. According to Stacey (2006), gay men as parents seem to challenge gendered and parenthood expectations more so than lesbian mothers. Gay men are judged more harshly than lesbians because they are perceived as violating traditional gender roles and the hegemonic model of masculinity (Connell, 2005; Wells, 2011). Heterosexual men and women hold more negative attitudes toward gay men than toward lesbians, reinforcing these sexual scripts (Herek, 2000). Consequently, gay men are perceived as being less capable of parenting well-adjusted children than lesbians (McLeod et al., 1999). In this sense, gay couples are evaluated by others as being less emotionally stable, as having fewer parenting competences, and as creating an environment that is inadequate, and even harmful for children (Crawford and Solliday, 1996).

In sharp contrast with these perceptions, a recent meta-analysis exploring the impact of gay fatherhood on children's psychological adjustment has found that in comparison with children of heterosexual parents, children of gay fathers may even fare better on some psychological domains, namely demonstrate less internalizing and externalizing problems (Miller et al., 2017). The authors argue that these differences may be attributed to better sociodemographic characteristics (e.g., higher income, level of education), as well as to the resilience shown by gay fathers in face of a discriminatory and oppressive social climate.

We have analyzed state-of-art research on gay fatherhood with the purpose of identifying the gaps in the literature, and highlight new and needed research avenues to pursue. In the last few years, at least three meta-analysis have been published. However, all of them have focused on the impact of gay/lesbian parenthood for child development (Allen and Burrell, 1997; Crowl et al., 2008; Fedewa et al., 2015). To our knowledge, no previous meta-analysis or systematic review has focused on parenting competences or family processes among gay and bisexual parented families. Furthermore, the few studies that have examined gay fatherhood are mostly qualitative, thus cannot be included and reviewed through meta-analytic procedures. We decided that a systematic and critical review would enable us to include both quantitative and qualitative studies in the sample of reviewed studies to provide a better understanding of the evolution and knowledge of the broader field of research on gay fathers.

## Materials and methods

### Search strategies

A comprehensive search of the relevant literature was undertaken using electronic databases (MEDLINE, PSYCHINFO, SCIELO, and GOOGLE SCHOLAR) and reference lists for articles published until December 2016. In addition, to identify other studies not easily accessible or less cited, expert researchers in the field were contacted. Further article searches were conducted in key reference journals in the field, namely the Journal of GLBT Family Studies and the Journal of Homosexuality. Searches included all possible combinations of the following terms: “gay,” “homosexual,” “same-gender,” “same-sex” with “father,” “parent^*^,” “famil^*^,” “adoption,” “surrogacy,” and “donor.”

### Inclusion/exclusion criteria

The main inclusion criterion was that articles reported original empirical data on gay fathers and/or children of gay fathers. A further criterion was that articles were published in peer-reviewed scientific journals until December of 2016. Exclusion criteria included review articles, studies with only lesbian parented families, and articles that simply combined lesbian-mother and gay- father families in one sample. Considering the dearth of studies about gay fatherhood, all studies including gay fathers and children of gay fathers were selected, regardless of family configuration, living arrangements, study design, or study objectives. The quality of the studies was not systematically assessed, as there was wide variation among them in terms of study designs, methods employed, sampling procedures, and, importantly, date of publication (1979–2016). However, a critical examination of the strengths and limitations of the studies was undertaken as part of the review. Our wide inclusion criteria enabled us to achieve a better overview of research on gay fathers and assess the state of the art in this sparse field.

### Coding of studies

A coding scheme based on the literature was developed to systematize the information collected from the reviewed studies. Two researchers separately coded the findings from each study, and the two coding schemes were compared and discussed. Inter-coder reliability was 90%, and a final list of themes was achieved after resolving the discrepancies.

## Results

### Descriptive overview of the studies

A total of 63 studies that fulfilled the inclusion criteria were collected (see list in Table 1). Studies spanned a publication period from 1979 to 2016, although almost three quarters of these were published after 2005, and close to 50% after 2011. The overwhelming majority of studies were conducted in the United States (68%). Noteworthy, five studies originated from Israel, and these were published after 2011. In Europe, five published studies were found: three of these from the United Kingdom, one from the Netherlands, and one from Denmark.

**Table 1:** Table of selected studies.

**Authors/date**	**Country**	***N* Fathers**	***N* Children**	**Fathers and/or children**	**Selection**	**Method**	**Methodology**	**Kinship**
Miller, 1979	CA/USA	40	90	Fathers and children	Convenience/purposive (gay community)	Qualitative	Interview	Mixed
Bozett, 1980	USA	18	0	Fathers	Convenience/purposive (gay community)	Qualitative	Interview	Mixed
Bozett, 1981	USA	18	0	Fathers	Convenience/purposive (gay community)	Qualitative	Interview	Heterosexual marriage
Robinson and Skeen, 1982	USA	60	0	Fathers	Convenience/purposive (gay community)	Quantitative	Questionnaire	Not reported
Skeen and Robinson, 1984	USA	30	0	Fathers	Convenience/purposive (gay community)	Quantitative	Questionnaire	Not reported
Skeen and Robinson, 1985	USA	60	0	Fathers	Convenience/purposive (gay community)	Quantitative	Questionnaire	Not reported (heterosexual)
Bigner and Jacobsen, 1989a	USA	66	0	Fathers	Convenience/purposive (gay community)	Quantitative	Questionnaire	not reported
Bigner and Jacobsen, 1989b	USA	66	0	Fathers	Convenience/purposive (gay community)	Quantitative	Questionnaire	not reported
Bigner and Jacobsen, 1992	USA	53	0	Fathers	Convenience/purposive (gay community)	Quantitative	Questionnaire	not reported
Crosbie-Burnett and Helmbrecht, 1993	USA	96	48	Fathers and children	Convenience/purposive + clinical referrals	Quantitative	Questionnaire	Heterosexual marriage
Bailey et al., 1995	USA	55	43	Fathers and children	Convenience/purposive (gay community)	Quantitative	Questionnaire	Not reported (heterosexual)
Peterson et al., 2000	USA	3	0	Fathers	Convenience/purposive (gay community)	Qualitative	Interview	Not reported (planned)
Barrett and Tasker, 2001	UK	101	179	Fathers and children	Convenience/purposive (gay community)	Quantitative	Questionnaire	Mixed
Silverstein et al., 2002	USA	21	0	Fathers	Convenience/purposive (gay community)	Qualitative	Focus group	Planned
Benson et al., 2005	USA	25	0	Fathers	Convenience/purposive (gay community)	Qualitative	Focus group	Heterosexual marriage
Current-Juretschko and Bigner, 2005	USA	5	0	Fathers	Convenience/purposive (gay community)	Qualitative	Interview	Step parenting
Schacher et al., 2005	USA	21	0	Fathers	Convenience/purposive (gay community)	Qualitative	Focus group	Heterosexual marriage
Stacey, 2006	USA	24	0	Fathers	Convenience/purposive (gay community)	Qualitative	Interview	Mixed
Berkowitz, 2008	USA	39	0	Fathers	Convenience/purposive (gay community)	Qualitative	Interview	Planned
Berkowitz and Marsiglio, 2007	USA	39	0	Fathers	Convenience/purposive (gay community)	Qualitative	Interview	Planned
Brinamen and Mitchell, 2008	USA	10	0	Fathers	Convenience/purposive (gay community)	Qualitative	Interview	Not reported (planned)
Gianino, 2008	USA	16	0	Fathers	Convenience/purposive (gay community)	Qualitative	Interview	Adoption
Riggs, 2008	AUS	21	0	Fathers	Convenience/purposive	Qualitative	Interview	Donor Insemination
Ripper, 2008	AUS	40	0	Fathers	National data file	Mixed-methods	Media analysis	Donor Insemination
Sirota, 2009	USA	0	136	Children	Convenience/purposive	Quantitative	Questionnaire	Heterosexual marriage
Downing et al., 2009	USA	64	0	Fathers	Adoption agencies	Qualitative	Interview	Adoption
Bos, 2010	NL	72	0	Fathers	Convenience/purposive (gay community)	Quantitative	Questionnaire	Planned
Tasker et al., 2010	USA	0	36	Children	Convenience/purposive (gay community)	Qualitative	Interview	Not reported
Tuazon-McCheyne, 2010	UK	13	0	Fathers	Convenience/purposive (gay community)	Qualitative	Focus group	Surrogacy
Patterson and Tornello, 2010	AUS	102	0	Fathers	Convenience/purposive (gay community)	Quantitative	Questionnaire	Mixed
Bergman et al., 2010	USA	40	0	Fathers	Surrogacy agencies	Qualitative	Interview	Surrogacy
Wells, 2011	USA	20	0	Fathers	Convenience/purposive (gay community)	Qualitative	Interview	Adoption
Berkowitz, 2011a	USA	^*^12	0	Fathers and non-fathers	Convenience/purposive (gay community)	Qualitative	Interview	Adoption
Berkowitz, 2011b	USA	^*^22	0	fathers and non-fathers	Convenience/purposive	Qualitative	Interview	Mixed
Armesto and Shapiro, 2011	USA	10	0	Fathers	Adoption agencies + support group	Qualitative	Interview	Adoption
Tornello et al., 2011	USA	230	0	Fathers	Convenience/purposive	Quantitative	Questionnaire	Adoption
Power et al., 2012	AUS / NZ	88	0	Fathers	Convenience/purposive	Mixed-methods	Questionnaire	Mixed
Richardson et al., 2012	USA	70	0	Fathers	Adoption agencies + convenience/purposive	Qualitative	Interview	Adoption
Goldberg et al., 2012a	USA	70	0	Fathers	Adoption agencies + convenience purposive	Qualitative	Interview	Adoption
Giesler, 2012	USA	12	0	Fathers	Convenience/purposive	Qualitative	Interview	Mixed
Tornello and Patterson, 2012	USA	167	0	Fathers	Convenience/purposive	Quantitative	Questionnaire	Heterosexual marriage
Dempsey, 2012a	AUS	6	0	Fathers	Convenience/purposive	Qualitative	Interview	Donor Insemination
Dempsey, 2012b	AUS	4	0	Fathers	Convenience/purposive	Qualitative	Interview	Donor Insemination
Lick et al., 2012	USA	0	86	Children	Convenience/purposive	Quantitative	Questionnaires	Mixed
Shenkman, 2012	IL	183	0	Fathers and non-fathers	Convenience/purposive (gay community)	Quantitative	Questionnaires	Mixed
Jenkins, 2013	USA	18	0	Fathers	Convenience/purposive	Qualitative	Interview	Heterosexual marriage
Dempsey, 2013	AUS	12	0	Fathers	Convenience/purposive (gay community)	Qualitative	Interview	Donor Insemination
Julien, 2013	CA	77	0	Fathers	Convenience/purposive	Quantitative	Questionnaire	Heterosexual marriage
Murphy, 2013	AUS / EUA	30	0	Fathers	Convenience/purposive	Qualitative	Interview	Surrogacy
Bucher, 2014	USA	50	50	Fathers and children	Convenience/purposive	Mixed	Interview	Heterosexual marriage
Erera and Segal-Engelchin, 2014	IL	9	0	Fathers	Convenience/purposive	Qualitative	Interview	Co-parenting with hetero women
Shenkman and Shmotkin, 2014	IL	204	0	Fathers and non-fathers	Convenience/purposive	Quantitative	Questionnaires	Mixed
Panozzo, 2015	USA	152	0	Fathers	Convenience/purposive	Quantitative	Questionnaires	Mixed (planned)
Tornello and Patterson, 2015	USA	739	0	Fathers	Convenience/purposive	Quantitative	Questionnaires	Mixed
Tornello et al., 2015a	USA	104	0	Fathers	Convenience/purposive	Quantitative	Questionnaires	Surrogacy
Tornello et al., 2015b	USA	511	0	Fathers	Convenience/purposive	Quantitative	Questionnaires	Mixed
Vinjamuri, 2015	USA	38	0	Fathers and non-fathers	Convenience/purposive	Qualitative	Interview	Adoption
Blake et al., 2016	UK	80	40	Fathers and children	Convenience/purposive and surrogacy agencies	Qualitative	Interview	Surrogacy
Erez and Shenkman, 2016	IL	180	0	Fathers	Convenience/purposive	Quantitative	Questionnaires	Mixed
Petersen, 2016	DK	15	0	Fathers and non-fathers	Convenience/purposive	Qualitative	Interview	Surrogacy
Perrin et al., 2016	USA	61	91	Fathers and children	Convenience/purposive	Quantitative	Questionnaires	Mixed
Shenkman and Shmotkin, 2016	IL	164	0	Fathers	Convenience/purposive	Quantitative	Questionnaires	Mixed (planned)
Vinjamuri, 2016	USA	38	0	Father	Convenience/purposive	Qualitative	Interview	Adoption

Of the total number of studies, 35 (56%) were qualitative (including interviews and focus groups), 25 (40%) were quantitative, and only three studies employed mixed-methods. Regarding data collection, the overwhelming majority of studies were based on convenience and/or purposive samples (91%), namely through contacts within the LGBT community. Looking at the samples, the studies collectively recorded 1,837 gay or bisexual fathers with 532 sons and daughters. Samples sizes varied between 3 and 739 fathers, parenting between 36 and 179 children. Most of the samples were composed by middle-class, highly educated, and predominantly white fathers, although compared with earlier research some recent studies with larger samples were able to capture a wider diversity in fathers' sociodemographic characteristics.

**Figure 1:**
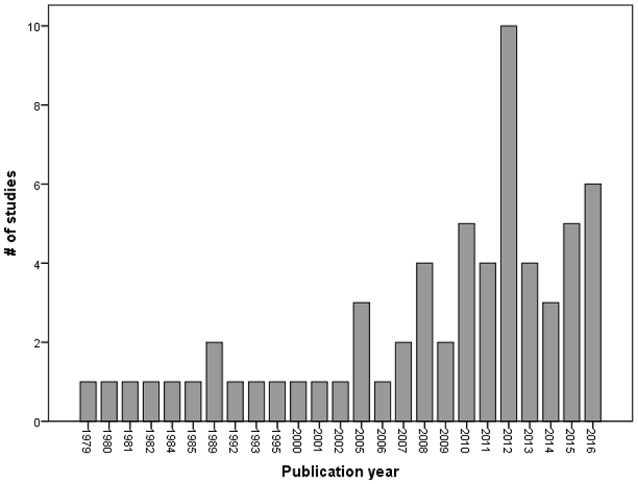
Number of published studies per year between 1979 and 2016.

A variety of parenting and kinship arrangements were identified in the samples studied. Slightly over a quarter of the studies recruited participants who had become fathers via diverse routes, and 16% were adoptive gay and bisexual fathers. Older studies focused on gay or bisexual fathers who had conceived their children within the context of a heterosexual relationship before coming out as gay or bisexual. More recent studies sampled a broader array of kinship arrangements involving gay and bisexual fathers, including adoption, surrogacy, step parenting, sperm donation to lesbian couples, and co-parenting arrangements with heterosexual women (see Figure 1).

Most of the studies (84%) focused only on fathers themselves, although some also enquired into children's development. We were able to group the research questions into the following nine themes to better review the reported findings: (1) Pathways to fatherhood; (2) Motivations for fatherhood; (3) Parenting experiences and childrearing; (4) Family life and relationship quality; (5) Gender and father identities and gender-role orientation; (6) Disclosure of sexual identity; (7) Social climate; (8) Father's psychosocial adjustment; and (9) Children's psychosocial adjustment. Notably, only one out of the nine research themes focused on children. Next, we review research under these themes of research questions.

### Pathways to fatherhood

“A generational change in timing and pathways to parenthood is taking place” (Tornello and Patterson, 2015, p. 44). The vast majority of older gay men became fathers in the context of heterosexual marriages prior to coming out, while younger gay men more often reported having children after coming out, namely through adoption, fostering or surrogacy (Patterson and Tornello, 2010; Tornello and Patterson, 2015). In a 2012 Australian study, the majority of the gay fathers (40%) became parents in the context of a previous heterosexual relationship, a smaller group (23%) via surrogacy arrangements, while 19% were donor fathers (in co-parenting arrangements with lesbian couples or single woman), and 11% had fostered their children (Power et al., 2012). One study on family membership revealed two different family patterns: *post-heterosexual divorce families* and *intentional families* (Jenkins, 2013). In the latter type of family, the gay fathers identified as their main difficulty institutional heterosexism in the form of non-recognition and religious condemnation of same-gender relationships, sometimes compounded by their ex-spouse in father-child relationship.

Regarding parenting through adoption, some studies revealed that men in same-gender couples experienced a more stressful pathway through this process than did heterosexual couples (Gianino, 2008; Berkowitz, 2011a). In fact, gay men who became fathers through adoption were confronted by additional layers of complexity because of the prevalence of heteronormativity and gendered norms. Downing et al. (2009) examined gay fathers' pathways to adoption in the U.S. and found that most couples (60%) had chosen to pursue private domestic open adoption. Another study revealed that very few agencies actively recruited gay adopters (Berkowitz, 2011a). Therefore, in order to be accepted as prospective adopter, many gay and bisexual men were forced to trust an informal and messy network of referrals (Wells, 2011). All of the men interviewed by Berkowitz and Marsiglio (2007) had considered adopting a child of a different race or ethnicity, and most of them described adoption as a very fatiguing financial and emotional process.

Semen donation and co-parenting with a (heterosexual or lesbian) woman also has offered gay men opportunities for creating a family (Erera and Segal-Engelchin, 2014). Gay and bisexual donors were found to be motivated to become donors because of their desire for parental involvement (Ripper, 2008). Conversely, donors in another study were described by the children's mothers as instrumental to conception, although not seen as fundamental for the children's lives (Riggs, 2008). One qualitative study identified different parenting arrangements between donors and their partners and lesbian mothers: Two men considered themselves as fathers whereas the third man saw himself as *just a donor* (Dempsey, 2012a). Those who considered themselves as fathers emphasized their previous desire to become a father from an early age, and highlighted the importance of a biological connection with their children. However, regardless of their own thoughts about their relationship with their children, their role was mainly decided upon by the child's resident mother(s) (Dempsey, 2012a,b). Two studies revealed that gay fathers in co-parenting arrangements with heterosexual women preferred this pathway to parenthood because they believed in the *essential mother* paradigm (Bucher, 2014). This belief meant that they valued the biological connection with their children, and believed that it was best for children to be raised by a mother and father (Bucher, 2014; Erera and Segal-Engelchin, 2014).

Similarly, gay fathers' choice of surrogacy as a pathway to parenthood was often rooted in the importance attributed to biological fatherhood (Petersen, 2016). Furthermore, gay couples reported knowing or wanting to know the biological father's identity between the two fathers (Dempsey, 2013). For some gay couples, the sperm donor was previously decided between them, and for other couples both of them provided the sperm, and only later discovered who the biological father was (Blake et al., 2016). Another study with planned gay father families reported that many gay couples went to great lengths to not reveal which father had the biogenetic connection with the child (Murphy, 2013). These men described three main strategies that they had employed to become fathers. One of these strategies was *turn taking* in deciding which partner would provide the sperm, so that one partner provided sperm for the first child, while the other partner would do so for the following pregnancy attempt. A second strategy was called *intentional unknowing* in which the eggs would be fertilized with the sperm of both partners and then multiple embryos would be transferred to the surrogate. A third strategy was maintaining *total secrecy*, in which both partners decided not to disclose which partner was in fact the biogenetic father.

Most gay fathers encountered complex ethical, practical, financial, and legal barriers to surrogacy. Tuazon-McCheyne (2010) found that Australian gay fathers using surrogacy had mostly international arrangements which meant most of these men returned home with their baby only to be challenged by legal barriers that made them feel anxious that they might lose legal custody of their children.

### Motivations for fatherhood

Regarding motivations for fatherhood several studies have shown that gay fathers have similar motivations for having children to those reported by heterosexual fathers, specifically, an *innate desire* for fatherhood. In fact, some of these men have made parenthood a *pivotal courtship criterion*, meaning that they considered fatherhood as the fundamental basis of their pursuit for love and intimacy. Regardless of their pathways to achieve parenthood (within different-gender or same-gender relationships) gay men referred to similar motivations across different studies (Bigner and Jacobsen, 1989a; Peterson et al., 2000; Stacey, 2006; Berkowitz and Marsiglio, 2007; Berkowitz, 2008; Goldberg et al., 2012a; Murphy, 2013). Studying planned families, Panozzo (2015) found that the gay fathers sampled presented an extremely high level of parenting desire.

### Parenting experiences and childrearing

Two studies reported that compared to heterosexuals, gay fathers go to greater lengths to enhance their children's cognitive skills, putting a greater emphasis on verbal communication and explaining rules (Bigner and Jacobsen, 1989b, 1992). Gay fathers also may be more emotionally expressive than more traditionally gender-role oriented heterosexual fathers. Nevertheless, when compared to heterosexual fathers, no significant differences were found regarding gay fathers in their involvement in their children's activities, level of intimacy with their children, parenting problem solving, time spent with their children, encouragement of children's autonomy, manner in which problems of childrearing were handled, emotional involvement with their children, and level of parental concern.

Among planned gay-father families with adopted children, parenting stress among gay fathers was within the normal range, well below clinical stress levels (Tornello et al., 2011). Another study sampling gay fathers in different planned kinship arrangements reported that gay fathers felt more insecure and less competent at childrearing than did heterosexual fathers in the comparison group (Bos, 2010). However, no differences were found between the gay and heterosexual father groups in relation to parental burden, parental concern, or levels of emotional involvement with their children.

### Family life and relationship quality

In a study with gay fathers and stepfathers, the main factor for family satisfaction was the inclusion of the stepfather into the father-child relationship, which was more important to family life than was financial comfort, family cohesion, or quality of the relationship with an ex-spouse (Crosbie-Burnett and Helmbrecht, 1993). Nevertheless, one study has indicated that some gay fathers have struggled with integrating their partner into their family life with their children (Jenkins, 2013). One study with post-heterosexual divorce gay fathers found that for the vast majority of (biologically related) children, gay fathers said they either shared post-divorce parenting with their ex-wife or at least had an important position in decision-making (Barrett and Tasker, 2001). In Barrett and Tasker U.K. study there were also differences between men in same-gender partnerships and those who were single regarding practical, material and emotional support, with partnered gay men experiencing fewer difficulties in parenting and more emotional and practical support compared to single gay fathers. Furthermore, Tornello and Patterson (2015) found that gay fathers in a same-gender relationship reported fewer identity issues, higher levels of perceived social support and self-disclosure, and lower levels of perceived stigma. Conversely, Giesler's study (2012) reported that the level of parenting stress experienced by gay fathers were independent of the father's relationship status.

With regard to the division of labor, two studies have reported that gay fathers tended to divide household and childrearing responsibilities equally; the greater the equality, the more satisfaction gay fathers indicated in their relationship (Tornello et al., 2015a,b). Furthermore, gay fathers who had achieved parenthood through surrogacy reported overall high levels of relationship quality.

Another two studies have indicated that most gay fathers shift their priorities with their child's birth, and experience changes in their work and lifestyle (Bergman et al., 2010; Richardson et al., 2012). Further, there were also changes in gay fathers' social lives, couple relationship and family relationship. For most of the fathers, having a child decreased the romance and personal intimacy with their partners. Furthermore, the frequency of gay fathers' social involvement diminished too; they tended to socialize more with heterosexual parents and reported having lost some gay friendships since becoming parents (Bergman et al., 2010). Another study revealed that gay fathers reported higher life satisfaction but gave less importance to their career since becoming a parent (Panozzo, 2015).

### Gender and father identities and gender-role orientation

In terms of gender and father identity and gender-role orientation, an early study with gay men who became fathers within heterosexual relationships reported no significant interaction between fatherhood status and fathers' endorsement of androgynous gender roles (Robinson and Skeen, 1982). In contrast, four studies published after 2000 have suggested a new paradigm of *degendered parenting* and *power sharing* in planned gay fatherhood (Silverstein et al., 2002; Schacher et al., 2005; Berkowitz, 2011b; Giesler, 2012). Thus, post-millennium planned gay fatherhood seems to promote a transformation of traditional masculine gender roles as these men assumed many different tasks and functions in childrearing that used to be associated solely with motherhood. Some fathers interviewed considered they had removed their parental role from their sense of gendered embodiment, while others thought they embodied both roles as mothers and fathers, extending the boundaries of what was accepted as *family* beyond biological connections (Berkowitz, 2011b).

In contrast, some gay fathers clearly still aspired to parenthood linked to the traditional “mother-nurturer, father-provider” family ideology (Berkowitz, 2011b; Panozzo, 2015). In an attempt to comprehend themselves as parents, some of the men in both studies identified with feminine parenting roles expectations, and framed their parenting experiences in maternal terms. Other authors have considered that gay men had to negotiate their parenting and family desires within a heteronormative societal system that was basically gendered, and therefore, tended to see motherhood as paramount in good parenting (Bucher, 2014; Erera and Segal-Engelchin, 2014).

Two studies have indicated that gay men often experience struggle in their journey to fatherhood (Gianino, 2008; Giesler, 2012). Gianino (2008) described six psychological steps gay men go through in deciding to embark on a journey to gay fatherhood: (a) abandoning a traditional parent identity, (b) finding comfort in a gay identity, (c) recognizing gay father families, (d) seeking models and mentors, (e) recognizing the strengths of being a gay father, and (f) articulating an expanded identity for themselves. Nevertheless, according to Armesto and Shapiro (2011) fathering for gay fathers catalyzed a reconstruction of their gay identity since parenting changed their lifestyle, and prompted their redefinition as both a gay man and a father. Fathering then emerged as the fundamental basis for their gay masculinity. In fact, gay fathers with an integrated gay identity tended to report higher perceived competence in parental tasks, higher levels of psychological adaptation, greater sense of personal growth, and greater contentment about being a parent compared to either gay fathers with non-integrated gay identities or heterosexual fathers (Julien, 2013; Shenkman and Shmotkin, 2016).

### Disclosure of sexual identity

In two studies about the sexual identity disclosure of gay men who had children in a heterosexual relationship, the majority of gay fathers described a difficult period of internal confusion as they tried to integrate their gay identity with their identity as a father (Bozett, 1981; Benson et al., 2005). In fact, many of them felt a severe identity conflict. Bozett (1980) reported that fathers' decisions about coming out to their children were based on their desire to preserve an honest and intimate relationship with them. In another study, disclosure seemed to promote father-child relationship to a higher level of emotional sharing (Benson et al., 2005). In contrast, in a 1980 study, fathers who did not disclose their sexual orientation reported self-rejection, and were very fearful of rejection (Bozett, 1980). They also referred to being afraid of possible vindictive behaviors from their ex-wife. These fathers pointed out two main reasons for disclosure: the necessity to explain the motives for divorce, or the need to explain and introduce their new relationship commitment to another man. In Bozett (1980) study, most fathers disclosed to their children by demonstrating their affection to another man in front of their children (indirect disclosure), and only subsequently talked directly with their children.

In a later UK study, Barrett and Tasker (2001) reported that the vast majority of children knew about their father's sexual orientation because most fathers reported that they had told their children directly. Children's mean age when they learned their father's sexual orientation was 11 years old. Tasker et al. (2010) concluded from children's interview data that for many their father's disclosure had been unexpected whereas for others it was a gradual process. The children then mostly made *selective disclosure* judgments in which they evaluated a particular social context with peers and when they considered them as safe, they would lift their usual protective frontier. As young adults, most recalled reaching a *nonchalant acceptance of* their father‘s sexual identity, and only a small number expressed any hostility and negative feelings specifically regarding the way their father expressed his sexual identity. Bucher (2014) has further reported that some gay sons grew concerned about their peers' perceptions of their masculinity because of their father's sexual identity.

In contrast to the generally more cautious approach to disclosure described by the gay fathers who came out of previous heterosexual relationships and their children, the approach of both fathers and children in planned gay-father parented families appears to be different. A study about planned fatherhood through adoption and surrogacy revealed that the fathers engaged in discussions about their “alternative family structure” with their children early on, while openly acknowledging their gay identities (Peterson et al., 2000). In a more recent study, partnered gay fathers who had made their coming-out before becoming a parent presented higher levels of disclosure in comparison to single gay fathers who became a parent prior to coming-out (Tornello and Patterson, 2015).

### Social climate

In Patterson and Tornello's (2010) study, it was suggested that gay fathers defied both personal and social conventions as they conquered fatherhood. After assuming and presenting their parenthood to the world, they faced discrimination from mainstream society and from other gay men. These gay fathers had overcame countless barriers to form their families. Nonetheless, they found opportunities to construct their own rules about child rearing and parenting, with their roles defined according to their personal competences and life circumstances. In fact, in two studies with adoptive gay fathers the vast majority reported being confronted with some level of inquiry or condemnation associated with their parenthood status, including from authority figures, thus being often reminded of the heteronormativity surrounding them (Vinjamuri, 2015, 2016). Further, gay fathers had faced unique challenges in making their adoptive and unconventional family as they anticipated and prepared themselves and their children for the discrimination they may encounter when revealing their family configuration. In this regard, a study of US gay fathers reported that in contrast with those living in California, most of the gay fathers who lived in Tennessee reported higher levels of parenthood-related stigma and tended to avoid situations in which they might encounter this stigma (Perrin et al., 2016) revealed.

### Father's psychosocial adjustment

In terms of the gay father's own childhood and upbringing, two studies indicated that the vast majority of gay fathers themselves had experienced an enjoyable childhood and adolescence (Skeen and Robinson 1984, 1985). Furthermore, in Skeen and Robinson's sample most gay fathers had grown up in families with both their mother and father present and seemed to appreciate stability of family relationships during their childhood. Further corroborating evidence was reported in Power et al.'s study (2012), in which the majority of gay fathers felt closely attached to their family-of-origin during adulthood, and reported regular contact with their parents.

In two recent studies, gay fathers presented higher levels of subjective well-being (positive emotions, judgments of life satisfaction and happiness) and a stronger sense of personal growth and purpose compared to both childless gay men and heterosexual fathers (Shenkman and Shmotkin, 2014, 2016; Erez and Shenkman, 2016). Another study found that gay stepfathers expressed the belief that they were not different from heterosexual stepfathers and families (Current-Juretschko and Bigner, 2005).

### Children's psychosocial adjustment

Two studies assessed the sexual orientation of youth with gay fathers (Miller, 1979; Bailey et al., 1995). According to these fathers, between 5 and 11% of their sons and daughters identified as lesbian, gay or bisexual, thus at a rate within general population estimates of sexual minority identification. Two other studies revealed that gay fathers perceived that their children did not exhibit emotional difficulties, hyperactivity or conduct problems (Barrett and Tasker, 2001; Bos, 2010). However, Barrett and Tasker (2001) pointed out three areas of concern that gay fathers reported having about their children's feelings and experiences, namely tension in keeping a secret, being teased or bullied by other children, and feeling different.

One study found that women with gay or bisexual fathers felt significantly less comfortable with nearness and intimacy, less able to trust others or depend on them to be available when needed, and also more anxious about their own intimate relationships than were women with heterosexual fathers (Sirota, 2009). The sample of women in Sirota's study were originally brought up in a heterosexual relationship, until their father later came out as gay. Thus, this study compared women who grew up in intact families with a heterosexual father and women who grew up in families whose parents divorced and whose father subsequently came out as gay. A further study has found a strong correlation between hegemonic masculinity and homophobia on sons of gay fathers (Bucher, 2014). Those who were more masculine tended to be less acceptant of their father's sexual identity and felt uncomfortable when telling others about their father's homosexuality.

For Lick et al. (2012), adult sons and daughters of gay fathers presented a positive psychological adjustment. Moreover, adult sons and daughters' well-being was shown to be associated with a positive social environment, even when they identified themselves as heterosexual. According to another study on adoptive gay parented families, fathers revealed that their children wanted to be open with others about their family configuration in spite of their fathers' fears about other children's reactions (Vinjamuri, 2016).

## Discussion

The central purpose of this review has been to highlight the gaps in research on gay and bisexual fathers in order to indicate novel and needed research directions. Studies about gay and bisexual fatherhood are still scarce (Golombok and Tasker, 2010) despite its exponential growth after 2005 and a further upturn in publications in 2012. As shown in this review, only 63 research papers were found that related specifically to the experiences of gay and bisexual fatherhood or considered the effects of having a gay or bisexual father on child and adolescent psychosocial adjustment. More than half of these studies were qualitative. Further, the large majority of studies were based on convenience or purposive samples, and most were conducted in the United States. The majority of the fathers in these studies were middle to upper-middle class, highly educated, and predominantly white, although recent studies have embraced more diverse sociodemographic samples. Very few studies included a comparison or control group (e.g., heterosexual fathers) or large and representative national samples. Thus, existing studies have been very heterogeneous regarding their goals, methods and methodologies, using questionnaires, interviews, focus groups, and media analysis, but the gay-father families sampled in the studies have been mostly homogeneous.

Underscoring the changing social context in which gay men can achieve fatherhood, older studies (conducted between 1979 and 2001) sampled gay and bisexual fathers who had children within a prior heterosexual relationship before coming-out. In contrast, contemporary studies have shed new light on diverse pathways to parenthood for gay men, namely through adoption, surrogacy, step parenting, co-parenting, and sperm donation (Tornello and Patterson, 2015). Nevertheless, gay fathers still point to the prevailing difficulties they encountered in planning a family. Gay men's conditions for childrearing are somewhat different from those of lesbian women not because of their sexual identity, but based upon ascribed gender roles, as it is still infrequent for fathers to be fully accepted as primary caregivers (Golombok et al., 2014).

Compared to the many studies of lesbian mothers that have mostly focused on their children's development, very few studies of gay or bisexual fathers have (see, for example, Tasker, 2005). Among the 63 studies, merely nine directly or indirectly evaluated the psychosexual development of children (adolescents and adults). As suggested by Golombok et al. (2014), child's psychological development and well-being have been studied almost exclusively in lesbian mothers' families. Regarding the sexual orientation of children of gay or bisexual fathers, it was not surprising that the percentages of those identifying as lesbian, gay or bisexual was within general population estimates (see, for example, Bailey et al., 1995). In fact, the vast majority of children raised in *non-traditional* families grow up to identify as heterosexual, despite having increased opportunities for consideration of different sexual identities (Tasker, 2005).

Regarding both gay fathers' and their children's psychosocial adjustment profiles, the reviewed studies have revealed that both fathers and children were generally well-adjusted. Gay fathers have reported that their children did not show any social, psychological or emotional problems (Barrett and Tasker, 2001; Bos, 2010). Fathers themselves also recalled generally pleasant childhoods, and were content on becoming parents (Skeen and Robinson, 1984, 1985). Moreover, gay fathers generally have reported high levels of well-being and satisfaction with life (Shenkman and Shmotkin, 2014; Erez and Shenkman, 2016). Nevertheless, fathers who had come-out after a heterosexual marriage tended to experience emotional turbulence when integrating their gay identity and their father identity (Bozett, 1980). However, the majority of Bozzett's fathers still chose to reveal their sexual identity to their children in order to secure or deepen their relationship with them. In contrast, for parents who planned parenthood in new family forms, the norm was for open and honest discussion about their family configuration from early on (Peterson et al., 2000).

Most intriguing is the way in which children of gay fathers seem to have accepted the revelation of their father's sexual identity in spite of public concerns about this. Some children already knew about their father's sexual identity, some were surprised, while for others it was a gradual process through which the overwhelming majority accepted and welcomed the revelation (Barrett and Tasker, 2001; Tasker et al., 2010). Nevertheless, Bucher's study (2014) reported that some sons might have struggled with accepting and disclosing their father's sexual orientation to others if the sons themselves were highly gendered and homonegative.

Regarding childrearing, gay and bisexual men clearly felt the need to justify their parental quality and efficacy, even in more recent studies. Since their effectiveness and capability as parents are constantly being questioned by society, gay and bisexual fathers like lesbian mothers may feel under more pressure to report a healthier parent–child relationship compared with heterosexual parents (Crowl et al., 2008; Gianino, 2008; Giesler, 2012). Nonetheless, in the studies reviewed, gay fathers did not show higher stress levels nor significant differences in their parenting skills when compared with heterosexual fathers (Tornello et al., 2011). However, gay fathers did indicate a greater investment in their children's activities and interests and higher levels of intimacy with their children, than did heterosexual fathers (Bigner and Jacobsen, 1989b, 1992). Furthermore, post-heterosexual divorce gay or bisexual fathers tended to report sharing parenting equally with their new same-gender partner if they did not remain single (Barrett and Tasker, 2001). Authors have previously suggested that in planning to have children together, gay couples may share parenting duties more equally than heterosexual couples, and the evidence on gay fathers parenting together also has indicated this (Bibarz and Stacey, 2010; Tornello et al., 2015a,b).

In terms of motivations for having children, gay fathers have reported similar motivations to heterosexual fathers, and a strong seemingly *innate desire* to become parents. Gay youth and young adults have indicated a high level of desire and motivation for parenthood (Riskind and Patterson, 2010; Baiocco et al., 2014; Panozzo, 2015; Costa and Bidell, 2017). However, there are many obstacles for gay and bisexual men in the pursuit of fatherhood, as the concept of fatherhood for gay men is highly influenced by societal prejudiced beliefs about gay men's gender and sexuality. In fact, it has been shown that not only societal gendered expectations and discrimination affects gay men's well-being and parenthood aspirations, but also internalized stigma has been associated with lower parenting desire among gay and bisexual men (Baiocco et al., 2014; Bauermeister, 2014).

Gay and bisexual fathers have suffered discrimination not only from society in general but also from other gay men (Patterson and Tornello, 2010). Discrimination has in turn led to a larger variety of pathways to parenthood because of the many obstacles to overcome in becoming a gay father (Costa et al., 2012). Parenting has been traditionally associated with femininity, and society in general considers women to be more nurturing and better suited for parenting than men (Anderssen et al., 2002). According to Berkowitz and Marsiglio (2007), men rearing children without the presence of a woman, regardless of their sexual identity, can be seen as contravening traditional masculine gender expectations, which can evoke discriminatory behaviors and negative attitudes. Moreover, stereotyped and negative beliefs about lesbians and gay men also call into question gay parent's capacities or skills (McLeod et al., 1999). Thus, children from families parented by gay or bisexual fathers might be exposed to higher levels of prejudice, compared with children in lesbian-parented families (Golombok and Tasker, 2010). Nevertheless, two-father families are becoming more visible and transforming the concept of *family* (Tornello and Patterson, 2015). Gay men are defying personal and social patterns in pursuing fatherhood (Silverstein et al., 2002), creating new opportunities, roles and guidelines about parenting and childrearing (Patterson and Tornello, 2010). Gay and bisexual fathers tend to demonstrate a wide variety of gendered positions that are sometimes very different from traditional conceptions of masculinity (Stacey, 2011). In public contexts (e.g., schools, workplaces, social spaces, etc.) gay fathers are likely to defy gender assumptions and this process will likely reflect on their children's own negotiation of conventionally gendered expectations (Hicks, 2013).

Furthermore, gay couples have faced more challenges in planning to have children in comparison to lesbian couples due to their reproductive circumstances. The literature on lesbian and gay parented families largely reflects families created through donor insemination by lesbian women. Other planned parenting arrangements such as adoption, fostering, or surrogacy require a greater effort from prospective parents (Patterson and Tornello, 2010), which may help to explain why there is a dearth of studies about these family configurations. Due to these many obstacles, the *gay baby boom* came much later and more slowly than the *lesbian baby boom* (Patterson, 1995). Since then, most literature has focused on child development and mothers' adjustment in lesbian-parented families, and the only study in this field with a longitudinal design has been with lesbian mothers who had their children through donor insemination (the National Longitudinal Lesbian Family Study [NLLFS]; Gartrell et al., 1996). This study has made it possible to evaluate how children grow up in a large sample of lesbian parented families since birth, which would be hard to replicate with gay parented families. In contrast, gay parenting is much more heterogeneous but remains underresearched. In this review, more than half of the studies have been published in the last few years, and the average number of published studies until 2009 was 1.4 per year. From 2010 onwards, the average number increased to 5.3.

### Methodological considerations and implications for future research

Comparative studies were necessary to demonstrate that children raised by lesbian and gay parents are not negatively affected in their psychosocial development by their parents' sexual orientation, and that lesbian and gay men are as capable as heterosexuals in parenting and childrearing (Crowl et al., 2008; Fedewa et al., 2015). Nevertheless, comparisons between the two types of families have reinforced heteronormativity, which undermines the particularities of lesbian and gay families; a wider concept of *family* needs to be further explored and discussed. Same-gender parented families and their children are often confronted with unique challenges due to social stigma (Stacey and Biblarz, 2001). Therefore, researchers should study the unique challenges and processes of same-gender parented families, particularly through qualitative and mixed methodologies that assess gay and lesbian parented families separately (Clarke, 2002). Our review deliberately included qualitative studies to allow direct access to the experiences and circumstances encountered by gay and bisexual fathers. Otherwise, it would have not been possible to observe the *degendered* redefinition of fathering, the process of sexual identity revelation and the process of normalizing same-gender relationships and family life.

Because there are fewer studies on gay parenting, one of the main criticisms of the field is focused on the non-representativeness of samples, particularly when considering the diversity in gay father family configurations (Tasker, 2005; Tasker and Patterson, 2008). We argue that given the financial, psychological and social resources needed to complete many adoption or surrogacy projects, (non-white) gay and bisexual men with lower income and educational level may not in fact be able to undertake these routes to parenthood. However, fathers who have taken other pathways to parenthood such as co-parenting with heterosexual or lesbian women may present different characteristics altogether. As Tasker (2013) has put it, “Future research should take into account the complex intersections of gender, sexuality, (dis) abilities, racial or ethnic differences” (p. 14).

Furthermore, the vast majority of the samples have been collected through convenience or purposive sampling, although some studies have now successfully recruited samples through assisted reproduction clinics and surrogacy and adoption agencies. Purposive and convenience sampling ought to be acknowledged as a methodological limitation on the representativeness of family configurations within gay father families. Nevertheless, despite the common use of convenience samples, our review showed that the findings were corroborated in different studies and in different countries, which is indicative of external validity.

Gay father populations are somewhat small but diverse which calls for a high level of methodological sophistication to overcome the barriers in sampling. Although, there is no conclusive evidence that gay men's pathways to parenthood are more diverse than those of heterosexual parents, researchers need to explore the qualitative differences inherent to this specific, extremely complex and non-homogeneous group. In short, researcher methodologies “will need to be considerably more refined than they have tended to be over the last 50 years” (Barrett and Tasker, 2001, p. 7).

Another important consideration regards children's psychosocial development. Few studies have explored the psychological and social adjustment of children raised by gay and bisexual fathers. One of the core aspects of child development is attachment, specifically the role of the main caregiver as a secure base who allows children to organize their behavior, feel secure and learn about their environment (Posada et al., 2004). Gay father's parenting in its essence is no different to that of lesbians or heterosexuals, with most studies demonstrating warmth and sensitivity on the part of gay father and the ability to build secure base relationships with their children, factors that in turn are predictive of children's psychological adjustment. The new planned family configurations such as families through donor insemination, surrogacy, and adoption by gay and bisexual men open up new possibilities to carry out naturalistic observations of children-father(s) exchanges since early childhood, which can yield important information that will broaden our understanding of developmental and family processes.

## Conclusion

Gay fathers are still seen more negatively and judged more harshly than lesbian mothers because society in general perceives them as undermining traditional gender roles. Consequently, gay men are perceived as being less capable of parenting well-adjusted children than are lesbians (McLeod et al., 1999; Herek, 2000; Wells, 2011). Conversely, according to Farr et al. (2010). Further evidence has highlighted that children of gay fathers may have even better outcomes than those of heterosexual parents in some psychological domains, namely less gender-stereotyped and less internalizing and externalizing behaviors (Goldberg et al., 2012b; Golombok et al., 2014; Miller et al., 2017). To date, most of what we know about the role of fathers in child development has been from research on heterosexual fathers parenting with mothers. Research on gay and bisexual fathers has given a particularly valuable opportunity to consider fatherhood per se in absence of motherhood.

## Author contributions

PAC, FAC, and IL: conception of the work; PAC and FAC: acquisition of data; PAC and FAC: data analysis; PAC, FAC, FS-Q, and FT: interpretation of data; PAC, FAC, FT, FS-Q, and IL: drafting the manuscript; final approval of the version to be published; agreement to be accountable for all aspects of the work.

### Conflict of interest statement

The authors declare that the research was conducted in the absence of any commercial or financial relationships that could be construed as a potential conflict of interest.
